# The Impact of Fitness Influencers on a Social Media Platform on Exercise Intention during the COVID-19 Pandemic: The Role of Parasocial Relationships

**DOI:** 10.3390/ijerph20021113

**Published:** 2023-01-08

**Authors:** Wenjia Li, Huangyi Ding, Guifen Xu, Jidong Yang

**Affiliations:** 1 College of Communication and Art Design, University of Shanghai for Science and Technology, Shanghai 200093, China; 2 School of Creativity and Art, Shanghai Tech University, Shanghai 201210, China

**Keywords:** social media, parasocial relationships, fitness influencer, exercise intention, COVID-19, personal attributes, fitness video

## Abstract

In the context of COVID-19 pandemic lockdowns, fitness influencers on social media are greatly involved in people’s home fitness processes, but there is limited research examining the role of fitness influencers on social media in exercise intention. This study aimed to explore whether people’s perceptions of the personal attributes and content quality of fitness influencers can promote a strong emotional connection between the two, thereby influencing people’s exercise intentions. Based on the theory of the parasocial relationship, we investigated the influence of social attractiveness (SA), physical attractiveness (PA), task attractiveness (TA), and content quality (CQ) of fitness influencers on the parasocial relationships (PSRs) between viewers and fitness influencers on social media and whether PSRs positively contribute to viewers’ exercise intentions (EI). The results revealed that SA, PA, TA, and CQ have positive influences on PSRs and CQ; PSRs directly contribute to EI; and PSRs have a mediating effect between EI and CQ. This study provides new insights into understanding the relationship between fitness influencers and people’s online fitness behaviors.

## 1. Introduction

The ongoing COVID-19 pandemic has disrupted people’s normal lives and changed their behaviors. Many countries imposed lockdowns, forcing people to stay at home to stop the spread of COVID-19. People around the world became increasingly active online, especially in terms of physical activity, as outdoor sports facilities and gyms closed, and it was recommended that people stay active by exercising at home [1]. An increasing number of people choose to exercise at home by following fitness influencers on social media. YouTube announced that the number of fitness videos compared with the previous year had increased more than five-fold during the 2020 pandemic [2], and it has been confirmed that many people in Australia and Germany used social media and other digital platforms for home workouts during lockdowns to maintain physical fitness [3,4]. In China, TikTok released a report in 2021 indicating that the cumulative number of likes on TikTok sports content videos exceeded 66 billion, and the number of sports creators with more than 10 thousand fans exceeded 56 thousand [5]. Fitness influencers are content creators on social media who provide people with free exercise skills and constantly interact with viewers in the process. They play a significant role in guiding and accompanying people in home-based workouts.

Social media may promote changes in physical activity behavior. In reviewing previous studies on social media and fitness, most of them focused on how the content posted on social media affects people’s attitudes and behaviors toward fitness. Body image on social media will exacerbate people’s social comparison and intentions to engage in extreme weight-loss behaviors [6]. Social media provides useful and interesting health information, which reinforces the perceived value of fitness and thus enhances people’s fitness intention [7]. By viewing exercise reports and fitness posts from friends on social media, individuals will be more motivated to change their exercise behavior [8]. Framed messages regarding mental health outcomes of physical activity via social media could be effective for physical activity motivation in universities [9]. There is not enough research on how fitness influencers affect people’s online fitness behavior. Studies have proven that fitness influencers positively affect people’s exercise intentions or behaviors [10]. Fitness influencers can be seen as health communicators on social media who use their professionalism, reliability, and attractiveness to motivate people’s fitness behavior [11]. The channel attributes and personal attributes of fitness influencers on YouTube will influence users’ behavioral intentions by affecting their flow experience and satisfaction [12].

However, the role of fitness influencers in people’s fitness behavior is still very unclear, considering their current characteristics and imagined intimate relationships through parasocial relationships. The focus of the present study is on how parasocial relationships between viewers and fitness influencers will impact people’s fitness intentions. Social media influencers (SMIs) represent a new type of independent third-party spokesperson who can shape audience attitudes through blogs, tweets, and the use of other social media [13]. The personal attributes of SMIs can attract viewers to interact with them and further develop into parasocial relationships (PSRs), which can persuade viewers to follow the attitudes and behaviors of the SMIs. While research on social media has explored the impact mechanism of PSRs on influencers and their followers in the marketing, fashion, and travel field [14,15], few studies have examined whether viewers and fitness influencers also have a psychological mechanism behind their PSRs in the field of fitness, and how this emotional connection affects viewers’ exercise intentions.

To further understand the impact mechanism between fitness influencers and users’ exercise intentions, this study empirically investigated what encourages users to build PSRs with their favorite fitness influencers on social media and how this impacts users’ exercise intentions. More specifically, we constructed a relationship model of social attractiveness (SA), physical attractiveness (PA), task attractiveness (TA), content quality (CQ), PSRs, and exercise intention (EI) based on parasocial relationships theory. This study expands the research on people’s online fitness behavior and reveals the mediating mechanism underlying the relationship between viewers’ EI and PSRs with fitness influencers.

## 2. Literature Review and Hypotheses

### 2.1. Parasocial Relationships

Horton and Wohl first conceptualized parasocial relationships as the intimate and one-way relationship between an audience and a media persona, which refers to interactions with media characters that are similar to real situations [16]. PSRs are built when audiences are frequently exposed to a persona and repeatedly have contact with them, which is similar to but different from real social interactions [16]. A related concept is parasocial interaction (PSI). PSI is audiences’ illusory and involved social experiences with media characters [16], which usually focus on short-term interactions between the two. A PSR is a more enduring relationship that a media user forms with a mediated performer. While PSI is restricted to the viewing episode, PSRs can extend beyond any single viewing episode [17].

Social psychological research proposes that PSRs include cognitive, emotional, and behavioral responses [18,19]. Prior studies have explored the dimensions of PSRs [17,18,19]. A PSR is a long-lasting process, and the media character’s attributes play a crucial role in the process. Media personalities’ interpersonal attraction should be considered an important dimension when considering the measurement of media personalities’ social relationships with audiences [20]. Interpersonal attraction usually includes three dimensions: physical attractiveness (PA), social attractiveness (SA), and task attractiveness (TA). PSRs are highly similar to real social relationships, so we can assume that the more interpersonal attraction a media character has with their audience, the easier it will be to form PSRs with them. This study set PA, SA, and TA were antecedents to PSRs. Social attractiveness refers to how socially likable a person is [14]. When individuals evaluate others, in addition to considering factors related to the ability dimension, factors related to the warmth dimension are also included in the evaluation category [21]. Specifically, when people judge whether a person is their friend during contact, they will evaluate whether he or she is friendly and sincere. Physical attractiveness refers to facial appearance or body image and is defined here as the degree to which a stimulus person’s image features are pleasing to observe [22]. The physical attractiveness of social media influencers positively influences PSRs between viewers and media personalities [23]. Task attractiveness refers to the attractiveness of social media influencers in terms of providing users with valuable and accurate information to help them complete their tasks [24]. When users encounter difficulties in life, they can rely on the information or services provided by media to complete tasks more efficiently, which increase their trust in media personalities, which in turn will promote the relationship between the two. Therefore, we propose the following three hypotheses:

**H1:**  *The social attractiveness of fitness influencers is positively related to viewers’ parasocial relationships*.

**H2:**  *The physical attractiveness of fitness influencers is positively related to viewers’ parasocial relationships.*

**H3:**  *The task attractiveness of fitness influencers is positively related to viewers’ parasocial relationships.*

### 2.2. Content Quality and Exercise Intentions

Content quality (CQ) refers to consumers’ perception of the accuracy, completeness, relevance, and timeliness of information created by influencers [25]. Entertainment and cognitive learning have been shown to be key to the satisfaction users seek from media consumption [26]. The core motivation for viewers to watch fitness videos on social media is to obtain fitness knowledge or skills, so the quality of fitness video content greatly affects users’ viewing behavior [27]. The formation of PSRs is an interpersonal engagement experience that requires a time investment, so the continuous consumption behavior of users on a digital influencer’s video content is conducive to the establishment of parasocial relationships between the two [28]. Therefore, this study proposes the following hypothesis:

**H4:**  *The content quality of fitness influencers positively influences the parasocial relationships between viewers and fitness influencers.*

Some studies emphasize the direct impact of CQ on individual intention [29]. Viewers believe that SMIs who provide high-quality content are reliable sources and are thus inclined to change their intentions or behavior when watching their content. Studies have shown that the value of social media influencers’ information positively influences followers’ trust in their brand posts, which in turn influences individual purchase intention and behavior [30]. The content quality of digital health information is also an important factor affecting users’ intention to continue using mHealth [3]. In the fitness field, which requires more professional content, the information quality of fitness influencers also has a greater impact on users’ intentions [12]. Based on previous studies, this study puts forward the following hypothesis:

**H5:**  *The content quality of fitness influencers positively affects viewers* *’ exercise intentions.*

### 2.3. Parasocial Relationships and Exercise Intentions

Originating from psychology, the PSR concept was at first mainly applied to traditional media such as television and broadcasting, and the idea has been gradually extended to new media studies with the development of technology. PSRs explain the phenomenon that influencers can greatly affect their followers’ behaviors and attitudes. Previous studies have explored the role of PSRs in the mechanisms by which social media influencers affect users’ intentions and behaviors. Specifically, in the field of marketing, Hwang found that PSRs between digital celebrities and their followers positively affected followers’ purchasing and electronic word-of-mouth (eWOM) intentions [31]. Lim also confirmed that a viewer’s wishful identification with an online video game streaming personality led to behavioral loyalty through the PSR with their favorite live streamer [32]. In the field of health, De Berail found that the intensity of the PSR with a favorite YouTuber was positively and significantly associated with the level of trust in that favorite YouTuber [33]. Sakib argued that weight-loss vloggers could influence viewers’ compliance intentions to follow healthy behaviors through the development of parasocial interactions [34], which may be more likely to predict users’ exercise intentions, as these are affected by PSRs with their favorite fitness influencers. At the same time, studies on social media have emphasized the mediating role of PSRs. Chung examined the mechanisms of social media use and their influence on the effectiveness of endorsers and found that PSRs have a mediating effect between social media interactions and source trustworthiness [35]. Blight made connections between virtual communities and parasocial relationships and found that Instagram users demonstrated the indirect effects of expressing motivation for information sharing and companionship from a sense of community through PSRs [36]. The results of these studies support a mediating role between PSRs and people’s perceptions, intentions, and behaviors on social media. Therefore, this study proposes the following hypothesis:

**H6:**  *The parasocial relationships between viewers and fitness influencers have a positive impact on viewers* *’ exercise intentions.*

**H7:**  *The parasocial relationships between viewers and fitness influencers mediate content quality and exercise intentions.*

**Figure 1:**
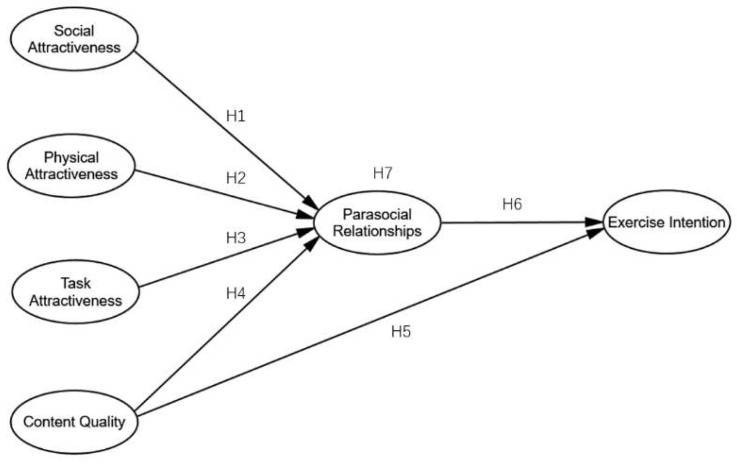
The research model.

From the discussion above, we proposed a conceptual model (see Figure 1).

## 3. Materials and Methods

This cross-sectional study was conducted to investigate the relationship between fitness intentions and fitness influencers on social media among a specific population isolated at home for the epidemic by questionnaire sampling during the period of the outbreak in China from March 2022-October 2022. We describe the distribution of healthy exercise during this time period and the relationship between personal attributes, content quality, prosocial relationships, and exercise intentions, establishing related hypotheses.

### 3.1. Sample and Procedures

An online survey was created using a reliable online survey platform (www.wjx.cn, accessed on 7 October 2022) and posted for Chinese people who actively viewed fitness videos on social media. The first item in the survey was a screening question, and potential participants were required to answer “yes” to participate in the survey (“Do you regularly follow fitness influencers on social media to exercise?”). We then set up an item to ask participants on which platform they followed the fitness influencer. The potential participants who answered “no” or listed an invalid answer were eliminated from the study data. The data were collected from 1 September 2022 to 6 October 2022, and a total of 494 questionnaires were collected. In this study, 29 answers to the first screening question were deleted, 5 answers were highly repetitive, and 44 invalid questionnaires with a very short duration were deleted, giving 416 valid questionnaires and an effective recovery rate of 84.2%. 

Table 1 shows the demographic characteristics of the participants, with 269 women (64.7%) and 147 men (35.3%) participating. The largest proportion of people was in the 18–28 age group (63.9%), and a smaller number were under 18 years old or over 60 years old. More than 60% of the participants had an education level above university. Of the total participants, 39.4% exercised 1–2 times a week, 35.8% exercised 1–2 times a week, 20.9% exercised 6–7 times a week, and only 3.9% exercised more than 7 times a week, and the proportion of those who exercised 1–2 h per week (38.5%) was slightly higher than the other two groups with shorter durations. Participants mentioned a total of 47 fitness influencers from China on social media, 19 of whom were male, and 28 were female. These fitness influencers are between the ages of 25–45. Among them, 36.2% have professional fitness instructor certification and provide more professional and comprehensive fitness content compared to others. 

### 3.2. Measures

There were six latent variables, and all the measurement items were adopted from a relevant maturity scale developed in previous research (see Appendix A Table A1). A 7-point Likert scale ranging from 1 (strongly disagree) to 7 (strongly agree) was used. Social attractiveness was assessed using four items from Greenwood (2008) [37]. Task attractiveness was assessed using the scale proposed by McCroskey (2006) [20]. We used a scale from Greenwood (2008) to measure the physical attractiveness of a fitness influencer [37]. The content quality measure was adapted from Kim Kim’s four items. PSRs were measured using the frequency scale proposed by Perse and Rubin (1988) [38]. Exercise intention was adapted and borrowed from Hsu and Lin (2008) and Durau (2022) [11,39].

### 3.3. Statistical Analysis

In this study, structural equation models (SEMs) were used to verify the hypotheses based on the study’s theoretical background. First, a descriptive demographic analysis of the sample was performed through SPSS 23.0 to analyze the general characteristics of the sample. Second, SPSS 23.0 and AMOS 24.0 were used for reliability analysis and confirmatory factor analysis to verify the rationality of the measurement model. Finally, AMOS 24.0 was used to test each hypothesis presented in this article. 

## 4. Results

### 4.1. Non-Response Bias

This study addresses the problem of non-response bias by comparing the gender and age of early respondents with those of later respondents. This approach is consistent with the procedure proposed by Armstrong and Overton (1977) [40], who argue that people who answer late are more like non-answerers than those who answer early [41]. Two hundred seventy respondents who completed the survey at an early stage were considered early respondents, and 146 respondents completed the survey at a later stage. The chi-square testing of early and late respondents showed no significant differences in gender and age (*p* > 0.05). Therefore, we excluded the possibility of no response bias.

### 4.2. Common Method Variance

To avoid common method bias, we controlled for the effect of common method bias by balancing the order of items, inserting different response direction items, and using anonymous questionnaires. We assessed the data set using controlling for the effects of an unmeasured latent factor method to identify any potential common method variance [42]. In addition to loading all questions measuring the constructs on their respective constructs, the same method latent factor was also loaded to compare whether the model fit was significantly better than the original model after controlling for the common method factor. When the common method factor was added to the original 6-factor model, the value of model fit indices (∆RMSEA = 0.013, ∆CFI = 0.018, and ∆IFI = 0.026) were improved below 0.03 [43], indicating that common method variance may not be a serious problem in the data set.

### 4.3. Assessment of the Measurement Model Reliability and Validity

This study followed the 2-step approach recommended by Anderson [44] to test the reliability and validity of the model. First, we analyzed the reliability of each construct using SPSS 23.0 software. The results showed that the Cronbach’s alpha values of all constructs were over 0.7 (see Table 2), exceeding the marginal level of reliability [45]. Second, this study confirmed the validity of all indicators through confirmatory factor analysis (CFA). The results were as follows: χ^2^ = 496.341, df = 218, (χ^2^/df) = 2.277, comparative fit index (CFI) = 0.963, the goodness of fit (GFI) = 0.898, normed fit index (NFI) = 0.936, Tucker–Lewis index (TLI) = 0.949, and root mean square error of approximation (RMSEA) = 0.055. This indicated that the model provided an acceptable fit for the data [46].

At the same time, convergent validity was examined in this study. As shown in Table 2, the standardized load for all latent variables ranged from 0.72–0.93, which was within the acceptable range (λ > 0.5) [47]. The composite reliability (CR) of all latent variables was greater than 0.8, and the average variance extracted (AVE) values were above 0.5 [47], which met the criteria of convergent validity. In order to test the discriminant validity, we compared the square root of the AVE with its correlations with any of the other constructs analyzed. From Table 3, it can be seen that the square root of the AVE of each latent variable was greater than its correlation with other latent variables, and the results met the criteria for distinguishing validity. Therefore, these results indicate that the measurement instrument in this study had good convergent validity and discriminant validity.

To further examine the multicollinearity problem, the variance inflation factor (VIF) test is performed on the main variables to further determine the severity of multicollinearity. VIF is used to test whether there is multicollinearity in the model; the larger the VIF, the more serious the multicollinearity problem. When the maximum VIF does not exceed five, the empirical rule considers that the model does not have a multicollinearity problem [48]. As Table 4 shows, the VIF of each variable is less than five, and the largest VIF value is 2.178, so the model is considered not to have the issue of multicollinearity.

### 4.4. Hypothesis Testing Results

#### 4.4.1. Main Effect Testing

**Figure 2:**
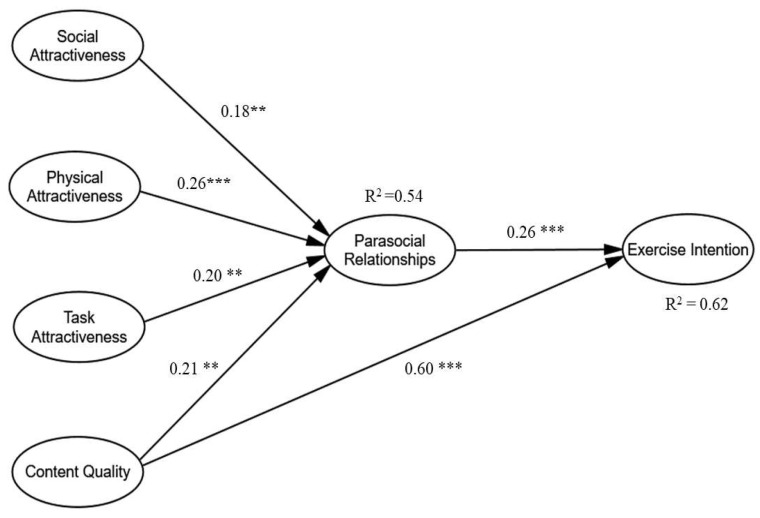
Results of structure model. ** *p* < 0.01; *** *p* < 0.001.

The relationships between variables were examined using the structural equation modeling (SEM) method. Figure 2 displays the results of the proposed model and the findings are presented in Table 5. The results indicated that PSRs are significantly affected by social attractiveness (β = 0.182, *p* < 0.01), physical attractiveness (β = 0.265, *p* < 0.001), task attractiveness (β = 0.198, *p* < 0.01), and content quality (β = 0.209, *p* < 0.001), suggesting that the higher social attractiveness, physical attractiveness, task attractiveness, and content quality fitness influencers have, the easier it is to build parasocial relationships with their followers. Hence, H1, H2, H3, and H4 were all supported. Content quality (β = 0.257, *p* < 0.001) significantly impacted exercise intentions, supporting H5. In addition, PSRs (β = 0.597, *p* < 0.001) significantly impacted exercise intentions, implying that the stronger the parasocial relationships people have with a fitness influencer, the stronger their intent to exercise. Therefore, H6 was supported.

#### 4.4.2. Mediating Effects

In order to explore the mediating effect of PSRs, this study used the bootstrap analysis method. At the 95% confidence level and with 2000 bootstrap samples, if the Z value was greater than 1.95 and there was no zero value within the 95% confidence interval, a mediation effect existed and was significant [49]. As shown in Table 6, the point estimate from CQ to EI was 0.056, the upper and lower bounds at the 95% confidence level did not contain 0, and the Z value = 2.240 > 1.96, which indicated that a mediating effect of PSRs between CA and EI existed; H7 was supported.

### 4.5. Regression Analyses and Significance Test

To further explore whether the number of workouts per week had an effect on the participants’ exercise intentions, we constructed a linear regression model and analyzed the relationship between the two. Due to exercise intention being measured by three items, we averaged the three items of the exercise intention variable. As shown in Table 7, the number of times per week that users follow fitness influencers to exercise (β = 0.278, *p* < 0.001) had a significant effect on their exercise intentions.

## 5. Discussion

This study provides a novel perspective on people’s home exercise behaviors based on social media in the context of the global pandemic. We constructed a conceptual model to investigate the relationships between personal attributes (SA, PA, and TA), content quality of fitness influencers’ videos, parasocial relationships and exercise intention. This study advanced the current literature by identifying viewers’ PSRs with fitness influencers as an important psychological mechanism that explains the effect of fitness influencers on viewers’ exercise intentions. The results of the study supported the research model well.

First, one major finding pertains to the role of content quality in shaping followers’ PSRs with fitness influencers, which has been less explored in previous research on the antecedents of PSRs. Previous studies have tended to focus more on the influence of personal attributes of influencers on viewers’ perceptions when defining the antecedents of parasocial relationships. However, social media influencers are important content creators, and there is evidence that the quality of content produced by social media influencers have a significant impact on the emotional attachment of their followers to them [50]. When viewers perceive fitness influencers’ content to be of high quality, they have a stronger emotional stickiness with the influencer. Additionally, the results of this study showed that social attractiveness, physical attractiveness, and task attractiveness of fitness influencers all positively and significantly contributed to PSRs. This is consistent with some previous literature, and most studies have shown that when viewers perceive positive attributes in media personalities, such as physical attractiveness, social attractiveness, and task attractiveness, they interact more with them, thus deepening the emotional trust between them and further developing parasocial relationships [51]. People were more inclined to find an encouraging and companionable workout partner during the period of lockdown, so influencers with strong social skills were more likely to be loved. It is noteworthy that PA facilitated PSRs more than SA and TA. Previous research on fitness has also highlighted the importance of the physical attractiveness of fitness instructors [52]. This suggests that viewers care more about the appearance and body of fitness influencers than other factors when watching online fitness videos.

Second, there was a significant positive correlation between content quality and viewers’ exercise intentions. Indeed, several studies have underlined this result. McClure found that the quality of information on social media positively influences the level of user engagement with a brand on social media [53]. Onofrei also confirmed the significant positive effect of content quality in terms of users’ purchase intentions [54]. According to perceived value theory, quality value is one of the most important dimensions [7], especially in the field of fitness, which is extremely demanding in terms of content. When people lost access to professional fitness instruction from gyms during lockdowns, they sought positive health information from social media. If they believed the content provided by that fitness influencer was professional, reliable, and useful, they would tend to follow their practice over time.

Finally, PSRs significantly and positively influenced viewers’ exercise intentions. In the health field, previous research has demonstrated that viewer-to-influencer PSI positively affects exercise intention [55]. We also found a mediating role of PSRs between content quality and exercise intention, which further emphasized the importance of parasocial relationships. According to social cognitive theory, the environment is an important factor influencing an individual’s cognition, and an individual’s interaction with the environment and others affects his or her cognition, emotion, and behavior [56]. From the previous results, it is clear that a fitness influencer who has a positive appearance, a strong ability to complete tasks, and engages in more interactions will be better able to construct interpersonal relationships with viewers that have an identity, connection, and engagement. At the same time, the content created by fitness influencers provides a complete information environment for users to easily and quickly find the fitness information they need. Therefore, we can further understand people’s motivation to follow fitness influencers on social media; on the one hand, people are driven by cognition and value the quality of content provided by fitness influencers. On the other hand, they also pay close attention to the companionship experience and emotional value brought by fitness influencers. Based on the emotional connection of PSRs, people will identify more strongly with the lifestyle ideas prompted by fitness influencers, thereby developing more exercise intention.

### 5.1. Theoretical Contribution

Our study focuses on the intimate relationships between viewers and fitness influencers, adding knowledge to the recent research stream of online fitness behaviors by investigating the combined effects of parasocial relationships on viewers’ exercise intentions. To explain the influential mechanism, we applied parasocial relationships theory and collected data from 416 Chinese who exercised via social media. The results indicated that the personal attributes and content quality of fitness influencers increase viewers’ parasocial relationships with influencers and, in turn, enhance viewers’ exercise intentions. This study provides new insight into how fitness influencers impact people’s exercise intention. 

Second, our study has some theoretical implications for parasocial relationships. Prior studies related to parasocial relationships focused on the marketing field; we further expand the use of parasocial relationship theory by verifying the practicality of this theory in the fitness field.

### 5.2. Practical Contribution

From the practical perspective, the study results provide some guidance on how fitness influencers on social media can increase their influence and thus change viewers’ fitness habits. First, fitness influencers should increase their personal attractiveness, thus strengthening the parasocial relationship with their followers. This intimate relationship strengthens the viewer’s trust in the fitness influencer and changes their attitude toward fitness. Our study reveals that the personal attributes of fitness influencers have a positive impact on viewers’ parasocial relationships, which in turn positively influences their fitness intention. Fitness influencers should actively engage in social interactions with the viewer, such as responding to comments on a regular basis and adding encouraging words to their fitness videos to increase communication with users. Also, fitness influencers should present their body image as perfectly as possible to improve their physical attractiveness, as users may be more inclined to follow fitness influencers who are good-looking.

Second, fitness influencers on social media should focus on improving the quality of content about exercise and fitness and becoming credible digital health communicators. Our results proved the importance of content quality. People search for fitness-related content on social media primarily to satisfy their information needs. Therefore, fitness influencers, as digital health communicators, should improve the quality of their content, such as working with professional coaches or doctors to create reliable, useful, and interesting home fitness videos that provide users with scientific and reasonable fitness guidance and programs. At the same time, in order to better popularize the awareness of fitness for all, fitness influencers on social media should appropriately lower the threshold for people to participate in fitness when producing fitness content, such as lowering the difficulty of fitness movements and simplifying the facilities used in fitness, so as to expand the range of applicability of fitness content.

## 6. Conclusions

This study aimed to investigate the effects of influencer attractiveness attributes and content quality on viewers’ parasocial relationships and whether PSRs further influence their fitness intentions. The findings demonstrated a positive link between PSRs and fitness intentions, validated the applicability of PSRs in the context of fitness behavior scenarios on social media, and enriched the perception of people’s online physical activities from a behavioral psychology perspective. According to the results of this study, SA, PA, TA, and CQ all play positive roles in establishing PSRs between viewers and fitness influencers. In turn, PSRs positively influence EI. Unlike previous efforts to understand people’s physical activities in terms of health consciousness, self-image, and other motivations, our results reflected that people’s emotional perceptions and intimate relationships with fitness influencers also motivate them to exercise. Therefore, we conclude that influencers on social media should focus on improving the quality of their content about exercise and fitness in order to become reliable digital health communicators, as well as enhancing personal attractiveness attributes to strengthen their emotional connection with viewers and better promote their home-based workouts. At the same time, people who want to keep exercising during lockdowns should try to interact with fitness influencers and further develop PSRs with their favorite influencers, thereby increasing their motivation to exercise at home in order to maintain better health levels.

## 7. Limitations and Future Research

Several limitations of this study might provide some suggestions for further research. First, the sample used in this study was limited to 416 online fitness workout users from Chinese social media, raising concerns about the generalizability of the results obtained. We created an online survey and used nonprobability sampling to collect data, which not only helped us to find potential participants on a larger scale to a certain extent but also inevitably caused some subjectivity. We encourage future research to circumvent these subjectivities and to obtain larger sample sizes to ensure the generalizability of the findings. 

Second, this study adopted a cross-sectional survey, and most of the answers were collected during the COVID-19 pandemic, lacking comparison to a typical period unaffected by the pandemic. Due to the cross-sectional nature of the study, temporal changes may occur in the user’s relationship with fitness influencers on social media and may affect their exercise intentions. Future longitudinal studies will help to determine whether the relationships between users and fitness influencers changed over time or if additional factors emerge that impact users’ exercise attitudes and intentions.

Third, this study only considered the personal attributes of fitness influencers and content quality as antecedents of parasocial relationships, but there may be other antecedents that influence parasocial relationships, such as wishful identification [32], the entertainment value of content [26], and so on. Apart from PSRs and content quality, perhaps there are other potential variables impacting users’ exercise intention, such as gender [11] and body image. Future research should further explore these ideas. We focused on the influence of fitness influencers on social media on people’s exercise intentions and verified the positive influence of fitness influencers on people’s exercise intentions from the psychological perspective of PSRs. Future research could go further and explore the impact on people’s actual fitness behaviors.

Finally, this article is based on the Chinese context, with fitness influencers and fitness users on mainstream Chinese social media (e.g., Weibo and TikTok) as examples. Future research could be extended to a global context to model the influence of fitness influencers on international social media on the exercise intentions of their followers.

## Figures and Tables

**Table 1:** Demographic profile of the survey participants (*N* = 416).

Variables (*N* = 416)	Category	Frequency
Gender	Male	147 (35.3%)
	Female	269 (64.7%)
Age	Below 18	19 (4.6%)
	18–28	266 (63.9%)
	29–45	87 (20.9%)
	46–60	30 (7.2%)
	Over 60	14 (3.4%)
Education	High school and below	140 (33.6%)
	Bachelor’s or college degree	168 (40.4%)
	Master’s degree or above	106 (26.0%)
Number of exercises per week	1–2 times	164 (39.4%)
	3–5 times	149 (35.8%)
	6–7 times	87 (20.9%)
	More than 7 times	16 (3.9%)
Number of hours per workout	Less than 1 h	112 (26.9%)
	Between 1 h and 2 h	160 (38.5%)
	More than 2 h	144 (34.6%)

**Table 2:** Reliability and convergent validity test.

Construct	Item	Ste	S.E.	Cα	AVE	CR
Social Attractiveness	SA1	0.784	-	0.899	0.697	0.902
	SA2	0.827	0.052			
	SA3	0.799	0.056			
	SA4	0.923	0.052			
Physical Attractiveness	PA1	0.830	-	0.890	0.733	0.892
	PA2	0.828	0.052			
	PA3	0.908	0.050			
Task Attractiveness	TA1	0.828	-	0.907	0.711	0.908
	TA2	0.808	0.048			
	TA3	0.836	0.049			
	TA4	0.899	0.046			
Content Quality	CQ1	0.804	-	0.915	0.729	0.915
	CQ2	0.833	0.055			
	CQ3	0.837	0.053			
	CQ4	0.936	0.052			
Parasocial Relationships	PSRs1	0.870	-	0.905	0.656	0.905
	PSRs2	0.815	0.049			
	PSRs3	0.819	0049			
	PSRs4	0.796	0.052			
	PSRs5	0.744	0.057			
Excise Intention	EI1	0.880	-	0.892	0.733	0.892
	EI2	0.869	0.044			
	EI3	0.819	0.047			

**Table 3:** Discriminant validity test.

	1	2	3	4	5	6
1. Content Quality	0.85					
2. Task Attractiveness	0.71	0.84				
3. Physical Attractiveness	0.64	0.64	0.86			
4. Social Attractiveness	0.67	0.68	0.58	0.83		
5. Parasocial Relationships	0.64	0.64	0.63	0.61	0.81	
6. Excise Intention	0.76	0.59	0.54	0.56	0.64	0.86

**Table 4:** Variance inflation factor (VIF) test.

	VIF	1/VIF
Parasocial Relationship	1.808	0.553
Content Quality	2.143	0.467
Task Attractiveness	2.178	0.459
Physical Attractiveness	1.813	0.552
Social Attractiveness	1.938	0.516

**Table 5:** Hypotheses testing.

	Hypothesis	β	*t*-Value	*p*	Result
H1	Social attractiveness→Parasocial Relationships	0.182	2.929	0.003	Support
H2	Physical attractiveness→Parasocial Relationships	0.265	4.452	***	Support
H3	Task attractiveness→Parasocial Relationships	0.198	2.849	0.004	Support
H4	Content quality→Parasocial Relationships	0.209	3.091	0.002	Support
H5	Content quality→Excise Intention	0.257	4.820	***	Support
H6	Parasocial Relationships→Excise Intention	0.597	10.471	***	Support

Note: *** *p* < 0.001.

**Table 6:** Unstandardized direct, indirect, and total effects of the hypothesized model.

CQ-PSRs-EI	Point Estimate	Product of Coefficients		Bootstrapping				Two-Tailed Significance
				Bias-Corrected 95% CI		Percentile 95% CI		
		SE	Z	Lower	Upper	Lower	Upper	
Direct Effects	0.625	0.069	9.058	0.053	0.389	0.387	0.052	0.001 **
Indirect Effects	0.056	0.025	2.240	0.016	0.121	0.012	0.112	0.004 **
Total Effects	0.681	0.064	10.641	0.053	0.389	0.052	0.387	0.001 **

Note: Unstandardized estimation of 2000 bootstrap samples, ** *p* < 0.01.

**Table 7:** Regression analyses and significance test (*N* = 416).

Model 1	Regression Analyses				Collinearity Statistics	
	B	S.E.	β	*p*	1/VIF	VIF
(constant)	4.798	0.131	-	0.000	-	-
Number of exercises per week	0.370	0.063	0.278	0.000	1.000	1.000

Note: Dependent variable: exercise intention, R^2^ = 0.077.

## Data Availability

Upon reasonable request, data used and analyzed during the current study are available from the corresponding author.

## Appendix A

**Table A1:** Scale of the study.

Construct	Item	Content
Social Attractiveness	SA1	This fitness influencer is easy to get along with
	SA2	I would like to have a friendly chat with this fitness influencer
	SA3	This fitness influencer would be pleasant to be with
	SA4	This fitness influencer is sociable with me
Physical Attractiveness	PA1	I think this fitness influencer is quite pretty (or handsome)
	PA2	I think this fitness influencer is sexy
	PA3	I find this fitness influencer physically attractive
Task Attractiveness	TA1	I would enjoy doing exercise with this fitness influencer
	TA2	I would recommend her/him as a fitness partner
	TA3	If I wanted to do exercise, I could probably depend on her/him
	TA4	I have confidence in this fitness influencer to get the job done
Content Quality	CQ1	This fitness influencer provides complete information on fitness
	CQ2	This fitness influencer provides useful information on fitness.
	CQ3	This fitness influencer provides timely information on fitness
	CQ4	This fitness influencer provides reliable information on fitness
Parasocial Relationships	PSRs1	He/she makes me feel comfortable, as if I am with friends
	PSRs2	If he/she appeared on another media, I would watch it to know more
	PSRs3	I look forward to watching the last video uploaded by him/she
	PSRs4	I miss seeing him/she when he or she is not publishing videos
	PSRs5	I would like to meet this fitness influencer in reality
Excise Intention	EI1	I intend to exercise with him/her within the next month.
	EI2	It is likely that I will exercise with him/her within the next month
	EI3	In the future, I would prefer to exercise to get healthy
